# Why We Disclose on Social Media? Towards a Dual-Pathway Model

**DOI:** 10.3390/bs15040547

**Published:** 2025-04-18

**Authors:** Qiyu Bai, Qi Dan, Yumin Choi, Siyang Luo

**Affiliations:** 1 School of New Media, Peking University, Beijing 100871, China; baiqiyu@pku.edu.cn (Q.B.); 2401213614@stu.pku.edu.cn (Y.C.); 2 Government Offices Administration of Chengdu, Chengdu 610042, China; danqi1998@163.com; 3 Department of Psychology, Sun Yat-Sen University, Guangzhou 510006, China

**Keywords:** social media self-disclosure, online social capital, loneliness, online interpersonal trust, Big Five, Chinese netizens, privacy paradox

## Abstract

Grounded in social penetration theory and social capital theory, this study aims to investigate how social media self-disclosure influences bridging and bonding online social capital, and how these in turn affect users’ loneliness and online interpersonal trust. A moderated mediation model was proposed and tested using cross-sectional survey data collected from 1519 Chinese netizens. Regression analyses revealed that self-disclosure on social media positively predicted both types of online social capital. Bridging social capital mediated the relationship between self-disclosure and reduced loneliness, while bonding social capital mediated the link between self-disclosure and enhanced online interpersonal trust. Moreover, agreeableness moderated the effect of self-disclosure on bonding social capital. These findings enrich the theoretical understanding of online self-disclosure and reveal the underlying motivations for users to disclose personal information on social media, even in the context of the privacy paradox.

## 1. Introduction

Social media refers to digital platforms that enable the creation and exchange of user-generated content through networked technologies (Kietzmann et al., 2011; Van Dijck & Poell, 2013). At present, it has become deeply integrated into people’s daily lives. Sharing personal information and expressing opinions and emotions on social media has become a widespread practice, commonly defined as social media self-disclosure (Bazarova & Choi, 2014).

In recent years, a growing body of research has examined social media self-disclosure. Most of these studies have approached the topic from the perspectives of social exchange theory and privacy calculus theory, focusing on privacy concerns, risk perception, and the protection of personal information on social platforms (Cheung et al., 2015; Gruzd & Hernández-García, 2018; Loiacono, 2015; Min & Kim, 2015; Tsay-Vogel et al., 2018). However, this line of research tends to overlook the positive motivations that drive individuals to disclose personal information online. As a core element in the development of interpersonal relationships, social media self-disclosure facilitates the formation of online social networks and can yield beneficial outcomes. Drawing on social capital theory, the present study investigates how social media self-disclosure influences two forms of online social capital: bridging social capital and bonding social capital. These two types differ in terms of relationship structure, member heterogeneity, and the nature of social support they offer (Putnam, 2000). Accordingly, this study proposes two moderated mediation models to examine how social media self-disclosure and individual personality traits contribute to the accumulation of these distinct forms of social capital, and in turn, how they affect individual loneliness and online interpersonal trust.

This study makes several contributions to the existing literature. First, it examines social media self-disclosure through the lens of social capital theory, exploring its relationship with online social capital and thereby enriching the theoretical understanding of self-disclosure behaviors on social media. Second, it differentiates between two distinct forms of online social capital—bridging and bonding social capital—and develops two mediation models to investigate their respective effects. Third, by incorporating agreeableness from the Big Five personality traits as a moderating variable, the study further uncovers individual differences in the accumulation of online social capital.

## 2. Literature Review and Research Hypothesis

### 2.1. Social Media Self-Disclosure and Online Social Capital

Self-disclosure refers to the act of revealing personal information to others (Jourard, 1964). Early research often conceptualized self-disclosure as verbal expressions of one’s thoughts, feelings, and personal information. However, with the development of social media, new technologies have enabled self-disclosure to occur in digital environments. Social media self-disclosure refers to the act of sharing personal information via social media platforms, such as posting about daily life or expressing personal opinions and emotions (Bazarova & Choi, 2014). According to social penetration theory (SPT), self-disclosure is the core of relationship development. Through the mutual exchange of private information, interpersonal relationships progress from superficial interactions to deeper emotional connections, ultimately fostering intimacy (Altman & Taylor, 1973). The establishment and accumulation of social relations form a social network, which enable individuals to connect with communities, obtain social support and build social capital.

Social capital refers to the resources existing in the social network that provide, support and facilitate goal attainment, especially in situations where such goals would be difficult to achieve without these connections (Coleman, 1988; N. Lin, 2002). Putnam classified social capital into two categories: bridging social capital and bonding social capital. Bridging social capital consists of relatively weak and inclusive ties that link individuals from diverse backgrounds, broaden their perspectives and grant access to novel resources and information. However, such relationships are broad in scope but low in depth, making them less effective for cultivating stable and intimate relationships. In contrast, bonding social capital involves stronger, more stable connections characterized by close interpersonal communication and emotional support. These relationships offer enduring mutual benefits but tend to exist within narrower, more homogeneous social circles (Putnam, 2000, 2004).

In its early conceptualizations, social capital primarily referred to offline social networks (Coleman, 1988; N. Lin & Dumin, 1986). With the development of technology—particularly the rise of social media platforms—studies have increasingly focused on online social capital (Cheng et al., 2019; Kaye et al., 2017; Phua et al., 2017; D. Williams, 2006). Similar to its offline counterpart, online social capital exists within digital social networks, which can also be divided into bridging social capital and bonding social capital. The maintenance of online social relations can help individuals accumulate online social capital (D. Williams, 2006). Social media plays a pivotal role in fostering these online relationships (Steinfield et al., 2013; J. R. Williams, 2019). Empirical research shows that social media usage is positively associated with both forms of online social capital, facilitating their formation and maintenance (Burke et al., 2010; Steinfield et al., 2008; Valenzuela et al., 2009).

By integrating social penetration theory and social capital theory, recent research provides a dual-perspective explanation: self-disclosure serves as a psychological and relational mechanism that facilitates not only intimacy development but also the formation of social capital across virtual platforms (Low et al., 2022; Ali et al., 2023). Building on this view, self-disclosure on social media not only deepens interpersonal connections but also contributes to expanding one’s online social networks. A higher level of self-disclosure may enhance both bridging and bonding social capital in digital contexts. Therefore, we propose the following hypothesis.

**Hypothesis** **1.** *Social media self-disclosure is positively correlated with both bridging and bonding online social capital.*

### 2.2. Online Social Capital, Loneliness and Online Interpersonal Trust

Bridging online social capital is conceptually similar to what Granovetter referred to as “weak ties” (Granovetter, 1973). It represents relatively superficial connections that emphasize breadth rather than depth. Within such large, dispersed and heterogeneous networks, individuals can access a wide array of information and social resources (Ellison et al., 2007). Consequently, bridging online social capital enables individuals to broaden their perspectives, gain access to diverse information and engage in wider online interpersonal interactions—factors that may alleviate feelings of loneliness. Loneliness is a kind of negative emotional experience arising from social isolation, often triggered by a gap between one’s desire for social interaction and the actual level of engagement, such as lacking a social network or not feeling accepted by others (Russell et al., 1980; Weiss, 1973). Previous studies have shown that individuals experiencing high levels of loneliness are often situated at the edge of social networks (Cacioppo et al., 2009) and that bridging social capital may help mitigate loneliness (Cheng et al., 2019; Sohn et al., 2019). We suggest that, since loneliness often stems from insufficient social interaction, bridging online social capital—cultivated through self-disclosure on social media—can help individuals participate in broader interpersonal exchanges and integrate into online communities. As a result, the gap between desired and actual levels of social interaction may be narrowed, ultimately reducing loneliness. Thus, we propose the following hypothesis:

**Hypothesis** **2.** *Bridging online social capital mediates the relationship between social media self-disclosure and loneliness. Bridging online social capital established through self-disclosure on social media helps reduce individual loneliness.*

Bonding social capital is more cohesive, which emphasizes the intimacy and stability of interpersonal relationships. It can provide strong emotional support, sustained reciprocity and assistance (Putnam, 2000). Individuals in the bonding network generally have a high degree of homogeneity, with similar social status and demographic characteristics. Members know each other very well and are highly united (Grootaert et al., 2004; Putnam, 2004), so individuals can feel more belonging within the bonding social network and thus generate group identity and interpersonal trust. Bonding social capital in cyberspace has similar characteristics (Phua et al., 2017; D. Williams, 2006), which enables individuals to obtain sustained support and mutual benefit in cyberspace and form united interpersonal relationships with other members online, generating stable expectations for the reliability of others, and then resulting in a high level of online interpersonal trust. Online interpersonal trust is an expectancy held by an individual or a group that the word, promise, or verbal or written statement of another individual or group in cyberspace can be relied upon (Feng et al., 2004). Online interpersonal trust often generates from stable interpersonal communication and social support online. It is found that a rigid and tight community is an important facet of interpersonal trust formation (Young & Tseng, 2008). As a kind of intimate relationship network, the online bonding social capital accumulated through social media self-disclosure can provide continuous online interpersonal interaction and social support for individuals, so as to enhance interpersonal trust online. Therefore, Hypothesis 3 of this study is proposed:

**Hypothesis** **3.** *Bonding online social capital mediates the relationship between social media self-disclosure and online interpersonal trust. The bonding online social capital established through social media self-disclosure helps to enhance online interpersonal trust.*

### 2.3. The Moderating Role of Agreeableness

Social media self-disclosure can promote the accumulation of online social capital, but the accumulation process varies from person to person. Therefore, it is necessary to involve personality traits as moderating variables in the process of online social capital accumulation. Big Five is a classic way to divide personality into five categories: openness, extraversion, agreeableness, neuroticism and conscientiousness. Many studies have found that agreeableness is closely related to social media participation, self-disclosure and interpersonal relationship building (Jenkins-Guarnieri et al., 2012; Kim et al., 2013; D. Liu & Campbell, 2017). Therefore, this study suggests that agreeableness can moderate the relationship between social media self-disclosure and online social capital.

Agreeableness reflects an individual’s attitude towards others, which is characterized by trust, frankness, altruism, obedience, humility and empathy (Graziano & Eisenberg, 1997). A highly agreeable person is usually friendly, understanding, close to others and good at handling the relationship with others (Asendorpf & Wilpers, 1998; White et al., 2004). Because of these characteristics, individuals of high agreeableness are more likely to establish good interpersonal relationships with others and expand their social network both online and offline (Asendorpf & Wilpers, 1998; H.-C. Huang et al., 2018; Selden & Goodie, 2018; White et al., 2004). Therefore, highly agreeable individuals are more likely to generate online social capital through social media self-disclosure. Based on the above discussion, Hypothesis 4 is proposed:

**Hypothesis** **4.** *Agreeableness moderates the relationship between social media self-disclosure and online social capital.*

**Hypothesis** **4a.** *Agreeableness moderates the relationship between social media self-disclosure and bridging online social capital. Individuals with high agreeableness are more likely to accumulate bridging online social capital through social media self-disclosure, thus reducing loneliness.*

**Hypothesis** **4b.** *Agreeableness moderates the relationship between social media self-disclosure and bonding online social capital. Individuals with high agreeableness are more likely to accumulate bonding online social capital through social media self-disclosure, thus enhancing online interpersonal trust.*

**Figure 1:**
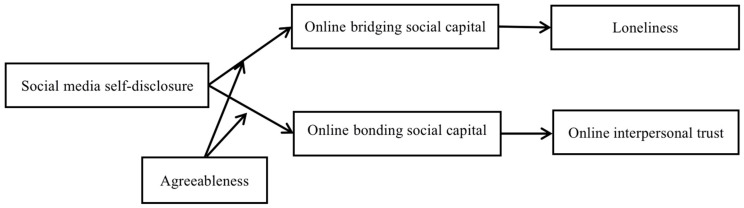
Research model.

To sum up, the overall research model of this study is shown in Figure 1:

## 3. Method

The primary objective of this study is to investigate the psychological and relational mechanisms underlying social media self-disclosure by examining its impact on two types of online social capital—bridging and bonding—and further exploring how these influence loneliness and online interpersonal trust. Drawing from social penetration theory and social capital theory, we proposed a dual-pathway moderated mediation model to test these mechanisms.

Specifically, Hypothesis 1 explores the direct relationship between social media self-disclosure and online social capital. Hypotheses 2 and 3 examine the mediating roles of bridging and bonding social capital in explaining the effects of self-disclosure on loneliness and online interpersonal trust, respectively. Hypotheses 4a and 4b investigate whether agreeableness, a core personality trait, moderates the first stage of these mediation pathways. These hypotheses are grounded in prior theoretical frameworks and aim to clarify how individuals psychologically and socially benefit from disclosing personal information in digital environments.

This integrated research design allows us to empirically test the relationships between the central constructs and provides a theoretically-informed explanation of social media users’ behavior in the context of the privacy paradox.

### 3.1. Participants and Procedure

The survey of this study was administered online via Wenjuanxing “https://www.wjx.cn” (accessed on 15 November 2021), a widely used Chinese survey platform. To enhance the representativeness of the sample, we utilized the filtering options available on Wenjuanxing to approximate the demographic and regional distribution of Chinese netizens. Although the sampling method was not strictly randomized, efforts were made to align the sample structure with the broader characteristics of China’s internet-using population.

The survey instrument was compiled based on widely used and validated scales from prior research. The questionnaire items were originally in English and were translated into Chinese using the back-translation procedure to ensure conceptual equivalence and accuracy. One scale—measuring online interpersonal trust—was originally developed in Chinese by two Chinese psychologists (Ding & Shen, 2005) and used directly in this study. The final questionnaire consisted of six sections corresponding to the core research variables: social media self-disclosure, online social capital (bridging and bonding), loneliness, online interpersonal trust, agreeableness and covariates (Appendix A).

The questionnaire was made available online for one month, during which time a total of 1600 responses were collected. An attention-check item was embedded in the survey to ensure data quality. After excluding responses that failed this item, a total of 1519 valid questionnaires were retained for analysis. All participants were Chinese netizens who voluntarily completed the survey and received a reward. The final sample was 77.3% female and 22.7% male, with an average age of 21.63 years (SD = 1.09). In terms of daily Internet usage, participants reported an average of 6.81 h online per day (SD = 2.82), with most spending between 4 to 10 h.

### 3.2. Measures

To examine the proposed hypotheses (H1–H4b), we used a structured questionnaire comprising six components, each corresponding to a core research variable in our model. These components included self-reported scales on social media self-disclosure, online bridging and bonding social capital, loneliness, online interpersonal trust and agreeableness. Each set of items was selected based on its conceptual alignment with the theoretical constructs and hypotheses. The following sections detail the measures used for each variable and their relationships with the relevant hypotheses.

#### 3.2.1. Social Media Self-Disclosure

The social media self-disclosure scale measures the level of individual self-disclosure on social media, including both the frequency and depth of self-disclosure. The scale is adapted from Krasnova’s social network self-disclosure scale (Krasnova et al., 2010). It consists of six items, including “when I have something to say, I am happy to post it on social media” and “I often update my profile on social media”. The scale was scored by 7 points. The higher the score was, the higher the degree of self-disclosure on social media was. Cronbach’s alpha for this scale was 0.87. This variable serves as the independent variable in our model and is involved in all four hypotheses (H1–H4b).

#### 3.2.2. Online Social Capital

To measure online social capital, this study adopts the two-dimensional online social capital scale developed by D. Williams (2006). There are 20 items in the scale. The first 10 items measure online bridging social capital, including items such as “Interacting with people online makes me interested in things that happen outside of my town” and “Talking with people online makes me curious about other places in the world”. The last 10 items measure online bonding social capital, with items including “There is someone online I can turn to for advice about making very important decisions” and “The people I interact with online would put their reputation on the line for me”. The scale was scored by 5 points (1 = totally disagree, 5 = totally agree). The Cronbach’s alpha coefficient of total scale was 0.89, that of bridging online social capital was 0.87, and that of bonding online social capital was 0.78. The two sub-dimensions—bridging and bonding online social capital—function as mediators in H2 and H3, respectively, and are also part of the moderated mediation model in H4a and H4b.

#### 3.2.3. Loneliness

The loneliness scale measures the loneliness caused by the gap between the desire for social interaction and the actual level. This study adopts the UCLA Loneliness Scale developed by Russell et al. (1980), which is composed of 20 items, such as “I feel in tune with the people around me” and “There is no one I can turn to”. Participants were asked to indicate how often they feel the way described in each of the statements on a four-point scale (1 = never, 4 = often). Cronbach’s alpha coefficient for this scale was 0.86. This variable is the outcome variable in H2, which posits that bridging online social capital, accumulated through self-disclosure, helps reduce loneliness.

#### 3.2.4. Online Interpersonal Trust

Online interpersonal trust refers to the reliability expectation of others online, which is established in the online interpersonal interaction. In this study, we used the interpersonal trust scale developed by two Chinese psychologists, Ding and Shen (2005). The scale consists of nine items with a five-point scale, except one, which is “how many trustworthy friends do you have on the Internet?” Items include “I believe that the information provided by most netizens is true” and “I am willing to share my secrets with other netizens and I know he or she is willing to listen”. In this study, the Cronbach’s alpha coefficient for this scale was 0.64. This variable is the dependent variable in H3, which hypothesizes that bonding online social capital mediates the effect of self-disclosure on online interpersonal trust.

#### 3.2.5. Agreeableness

In this study, agreeableness was measured by the Big Five personality scale (Saucier, 1994). There were 40 questions in the scale, and eight items for each personality. The participants chose some adjectives of personality traits in the five-point scale (1 = very true of me, 5 = not very true of me). In this study, the Cronbach’s alpha coefficient for agreeableness is 0.73. This variable is the moderator in H4a and H4b, examining how personality differences influence the indirect effects of self-disclosure on the outcome variables through online social capital.

#### 3.2.6. Covariates

Considering that time spent online will have some impact on individuals’ self-disclosure on social media and the acquisition of online social capital, the average daily online time is taken into the research model as a covariate. In addition, gender and age, as basic demographic characteristics, are also included in the research model as control variables. These covariates were statistically controlled in all models to isolate the primary effects related to the hypotheses.

### 3.3. Statistical Analyses

SPSS 26.0 was used for data analysis. In the first step, we performed descriptive statistical analysis, calculating the mean, standard deviation and correlation matrix of variables. In the second step, PROCESS macro Model 4 (Hayes, 2012) was used to test the mediating role of online bridging and bonding social capital. In the third step, the PROCESS macro Model 7 (Hayes, 2012) was used to test the moderating role of agreeableness. Finally, in order to test the significance of all the effects, the bootstrapping method was used, which resampled the data 5000 times and generated 95% bias-corrected confidence intervals of all the effects. If zero was not included in the confidence interval, effects were significant.

## 4. Results

The purpose of this study was to analyze the relationship between social media self-disclosure, loneliness and online interpersonal trust, as well as to explore the moderating effect of agreeableness. The data analysis was carried out by the following three steps:

### 4.1. Primary Analyses

Using SPSS 26.0, the descriptive statistical information and correlation matrix of variables were calculated. The results are presented in Table 1. The results show that social media self-disclosure is significantly positively correlated with online bridging social capital (r = 0.40, *p* < 0.001) and online bonding social capital (r = 0.41, *p* < 0.001), which verifies hypothesis 1. Online bridging social capital is negatively correlated with loneliness (r = −0.20, *p* < 0.001), while online bonding social capital is positively correlated with online interpersonal trust (r = 0.43, *p* < 0.001). In addition, agreeableness is positively correlated with online bridging social capital (r = 0.17, *p* < 0.001).

### 4.2. Testing for Mediation Effect of Online Social Capital

Drawing upon PROCESS macro Model 4, this study examines the mediating role of online bridging social capital and bonding social capital.

Regression results (as shown in Table 2) show that after controlling gender, age and average daily time spend online, social media self-disclosure significantly affects bridging network social capital (*β* = 0.21, *p* < 0.001. See Table 2, Model 1), and bridging network social capital significantly affects loneliness (*β* = −0.15, *p* < 0.001. See Table 2, Model 2). There is an indirect effect between social media self-disclosure and loneliness mediated by online bridging social capital. The significance of this indirect effect is verified by the bootstrapping results. The indirect effect is -0.03 with a 95% confidence interval of [−0.0425, −0.0222], which does not contain zero. Therefore, Hypothesis 2, that online bridging social capital mediates the relationship between social media self-disclosure and loneliness, is supported.

The result of testing the mediating effect of online bonding social capital (as shown in Table 3) shows that social media self-disclosure has a significant effect on online bonding social capital (*β* = 0.21, *p* < 0.001, see Table 3, Model 3), and there is a significant positive correlation between online bonding social capital and online interpersonal trust (*β* = 0.23, *p* < 0.001. See Table 3 Model 4). There is an indirect effect between social media self-disclosure and online interpersonal trust mediated by online bonding social capital, and its indirect effect is 0.05, with a 95% confidence interval of [0.0392, 0.0596], which does not include zero. Therefore, the mediating role of online bonding social capital between social media self-disclosure and online interpersonal trust has been verified, supporting Hypothesis 3.

### 4.3. Testing for the Moderation Effect of Agreeableness

According to Hypothesis 4, this study suggests that the influence of social media self-disclosure on loneliness and interpersonal trust through online social capital is moderated by agreeableness. We used PROCESS macro model 7 to examine this moderation effect. Results are shown in Table 4. The interaction between social media self-disclosure and agreeableness has a significant impact on online bonding social capital (*β* = 0.09, *p* < 0.01. see Table 4 Model 7). The index of moderated mediation is 0.0197, with a 95% confidence interval of [0.0039, 0.0363], excluding zero, which verifies that agreeableness moderates the indirect effect of social media self-disclosure on online interpersonal trust through online bonding social capital, supporting Hypothesis 4b. However, the interaction between social media self-disclosure and agreeableness has no significant impact on online bridging social capital (*β* = 0.03, *n.s.* see Table 4 Model 5), so Hypothesis 4a is not supported. Therefore, Hypothesis 4 is partially verified.

To sum up, agreeableness moderated the indirect relationship between social media self-disclosure and online interpersonal trust through online bonding social capital, while the moderating effect on the relationship between social media self-disclosure and loneliness via bridging social capital was not significant.

**Figure 2:**
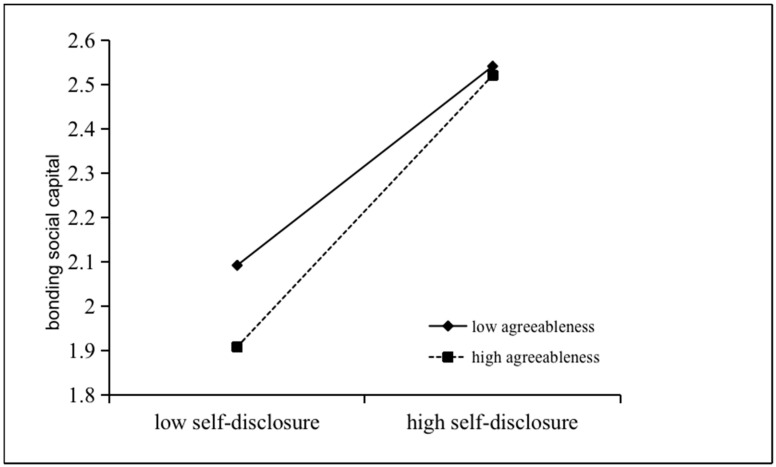
Interaction effect of social media self-disclosure and agreeableness on online bonding social capital.

In order to clarify the moderating role of agreeableness, simple slopes analysis is used to describe how the interaction between social media self-disclosure and agreeableness affects online bonding social capital. The moderating variables are divided into low and high levels (1 SD below the mean and 1 SD above the mean) and predicted online bonding social capital against social media self-disclosure is plotted, as shown in Figure 2.

The results show that social media self-disclosure has a significant impact on bonding social capital in both high and low agreeableness groups. In the high agreeableness group, the influence of social media self-disclosure on bonding social capital is stronger (b_high agreeableness_ = 0.25, t = 4.97, *p* < 0.001) than that in the low agreeableness group (b_high agreeableness_ = 0.18, t = 3.92, *p* < 0.001), which means that social media self-disclosure has a greater impact on the online bonding social capital of individuals with high agreeableness.

## 5. Discussion

### 5.1. Research Findings

This study aims to explore the impact of social media self-disclosure on two different types of online social capital. By constructing two moderated mediation models, this study finds that social media self-disclosure is significantly positively correlated with bridging and bonding online social capital, and has a positive impact on individuals’ psychological condition and interpersonal relationships, while personality traits moderated this process.

Specifically, this study finds that social media self-disclosure is positively correlated with bridging and bonding online social capital, with online bridging social capital moderating the relationship between social media self-disclosure and loneliness, while online bonding social capital moderates the link between social media self-disclosure and online interpersonal trust. This finding is consistent with social penetration theory, namely that in the cyber environment self-disclosure can also serve as the core of relationship development to promote the establishment of interpersonal networks and bring about positive results (H.-Y. Huang, 2016; Z. Liu et al., 2016). At the same time, according to social capital theory, social capital can be divided into bridging social capital and bonding social capital. They are of different characteristics and provide different social support to individuals (Patulny & Svendsen, 2007; Putnam, 2000). The results of this study verify the different effects of two kinds of online social capital: as a more extensive social network, online bridging social capital can provide more social connections for individuals, thus reducing loneliness, while online bonding social capital is a more intimate social relationship network, which can provide stable emotional support, thus improving online interpersonal trust.

In addition, the results also show that the relationship between social media self-disclosure and online bridging social capital is moderated by agreeableness, one of the Big Five personality qualities; that is, social media self-disclosure has different effects on online bonding social capital of individuals with different personalities. For individuals with high agreeableness, social media self-disclosure has a greater impact on their online bonding social capital, while individuals with low agreeableness are the opposite. This finding reveals that individual differences are important in the process of online bridging social capital accumulation, and that highly agreeable people are more likely to accumulate bonding social capital even in cyber space. However, contrary to the hypothesis, the moderating effect of agreeableness on the relationship between social media self-disclosure and online bridging social capital is not significant. This may be related to the characteristics of agreeableness itself. Agreeableness plays a more obvious role in establishing close and stable relationships with others, but it is not so prominent in building some shallow relationships.

### 5.2. Theoretical Contribution

This paper makes contributions to the existing research in the following aspects. Most of the existing studies of social media self-disclosure focus on questions such as privacy computing and risk management, exploring the influencing factors of online self-disclosure (Hallam & Zanella, 2017; Tsay-Vogel et al., 2018). It is found that online risk perception, privacy concerns, social media trust and other factors will hinder users’ self-disclosure (Choi & Bazarova, 2015; W.-Y. Lin et al., 2016; Tsay-Vogel et al., 2018). However, even if there are many risks in social media self-disclosure, users will not stop sharing information on the Internet, which is known as the “privacy paradox” (Barnes, 2006; Kokolakis, 2017). Researchers believe that “perceived benefit” is a key concept to explain the privacy paradox (Wilson & Valacich, 2012), but little is known about what benefits brought by online self-disclosure are the driving force of users’ self-disclosure. From the perspective of social penetration theory and social capital theory, this study explores the positive impact of social media self-disclosure, and finds that social media self-disclosure has a positive effect on individual psychological condition and interpersonal relationships, that is, helping individuals accumulate online bridging and bonding social capital, thus reducing loneliness and enhancing interpersonal trust. This discovery reveals the positive results of social media self-disclosure, enriches the research on online self-disclosure at the theoretical level, and provides a new perspective for understanding the social media self-disclosure behavior against the background of the “privacy paradox”.

Secondly, with the development of the Internet, cyberspace has become an important field to obtain social capital (D. Williams, 2006). This study introduces the concept of social capital into cyberspace, enriching the current research on online social capital. In addition, this paper further explores the different characteristics and influences of two types of online social capital: bridging social capital and bonding social capital. Because of its wide range and inclusiveness, bridging social capital can meet individuals’ social needs and reduce loneliness, while bonding social capital can provide stable social support because of its depth and intimacy, so as to enhance online interpersonal trust. The study of these two kinds of online social capital helps us to understand the influence path of social media self-disclosure on individual psychological conditions and interpersonal relationships.

Thirdly, this study offers theoretical contributions by clarifying the differential roles of bridging and bonding social capital in the context of social media self-disclosure. While previous research has often treated online social capital as a unified construct, our findings demonstrate that these two dimensions exert distinct positive psychological effects: bridging capital helps buffer loneliness by expanding weak-tie networks, whereas bonding capital strengthens interpersonal trust through closer relationships. This nuanced understanding advances both social capital theory and social penetration theory by illustrating how the quality of social ties mediates the relational outcomes of online disclosure. Furthermore, these findings provide new insight into the privacy paradox, revealing that the perceived relational benefits of self-disclosure—such as reduced loneliness and enhanced trust—may serve as intrinsic motivations for users to share personal information online, despite awareness of privacy risks.

### 5.3. Practical Implications

In practical terms, this study provides a reliable path for understanding the establishment of online interpersonal networks. With the development of social media, people’s social needs also need to be met in cyberspace, so it is increasingly important to establish and maintain interpersonal networks online (Chen, 2011; Davis, 2012). Social media self-disclosure is an important way to establish the online interpersonal relationship. Through social media self-disclosure, individuals can develop interactive relationships with others in cyberspace, build online relationship networks and accumulate bridging social capital and bonding social capital, so as to meet their social needs in cyberspace.

Secondly, this study provides new support for the practice of using social media to reduce individual loneliness. There has been a long-standing debate on the relationship between Internet use and loneliness. Some studies believe that online communication lacks emotional cues compares with face-to-face communication, and immersion in cyberspace replaces meaningful social interaction offline, which will result in loneliness (Kraut et al., 1998; Turkle, 2017). However, some studies believe that the characteristics of the Internet itself can stimulate the establishment of relationship networks, which can meet individuals’ social needs and provide information or emotional support, so as to reduce loneliness (S. Lin et al., 2020; Teppers et al., 2014; Valkenburg & Peter, 2007). This study verifies the latter hypothesis through empirical research. Through social media self-disclosure, individuals can expand their social network online, and this extensive network can reduce loneliness. Therefore, the interpersonal relationship in cyberspace should not be ignored; it can also meet individuals’ social needs.

Thirdly, the findings of this study point out a feasible way to enhance interpersonal trust and build a harmonious cyberspace. Although the Internet brings convenience to people’s communication, it also has the problems of anonymity and uncertainty, which brings risks to network communication (Livingstone & Helsper, 2007; Valkenburg & Peter, 2011). In this context, how to build a trusted Internet and enhance trust in network communication has become an urgent problem. This study found that an effective way to build online interpersonal trust is to establish deep and intimate bonding interpersonal networks through self-disclosure, so as to produce stable interpersonal communication and informational and emotional exchange, which will enhance online interpersonal trust and establish a harmonious and mutually supportive network space.

Last but not least, these findings have practical value for the field of marketing and online platform management: understanding the pathways through which self-disclosure affects social capital and trust can help improve user engagement strategies. For instance, platforms could design features that encourage meaningful and controlled self-disclosure, thereby fostering a sense of connection and trust among users. This is particularly useful for social networking services and digital content communities that aim to enhance user retention through emotional and social bonding. Furthermore, mental health interventions could leverage this knowledge by promoting positive self-disclosure practices that help users build supportive online networks, thereby reducing loneliness.

### 5.4. Limitations and Future Perspective

This study has several limitations. First, the use of a cross-sectional survey design limits the ability to draw causal inferences. While significant associations were found between social media self-disclosure and psychological or relational outcomes, it remains unclear whether self-disclosure is the driving force behind changes in online social capital, loneliness or interpersonal trust. Future studies employing longitudinal or experimental designs may help to verify these causal pathways.

Second, the study relies solely on self-reported questionnaire data, which may be subject to common method bias, social desirability and limited introspective accuracy. Although validated scales were used, combining self-reporting with behavioral data or qualitative interviews in future research would provide a more comprehensive understanding of the mechanisms involved.

Third, the sample consists entirely of Chinese netizens, which constrains the generalizability of the findings to other cultural and geographical contexts. Cultural norms around self-disclosure, privacy and online social interaction may differ substantially across regions. Future research should replicate this model in diverse socio-cultural settings to test its cross-cultural validity.

Finally, although this study focused on agreeableness due to its strong relevance to interpersonal processes, other personality traits (e.g., extraversion, neuroticism) may also play important roles in shaping how individuals accumulate online social capital. Future studies could adopt a broader personality framework to enrich the theoretical understanding of these dynamics.

## 6. Conclusions

This study focuses on the psychological and social outcomes of social media self-disclosure. Drawing on social penetration theory and social capital theory, we developed a dual-pathway moderated mediation model to examine how self-disclosure influences two types of online social capital—bridging and bonding—and how these, in turn, affect loneliness and online interpersonal trust. The findings demonstrate that social media self-disclosure positively contributes to both bridging and bonding social capital. Specifically, bridging social capital serves to reduce loneliness, while bonding social capital enhances interpersonal trust. Agreeableness was found to moderate the pathway involving bonding social capital but not bridging social capital.

Importantly, these findings offer a novel perspective on the longstanding “privacy paradox”—the phenomenon in which individuals continue to disclose personal information on social media despite concerns about privacy. While previous studies have highlighted risk–benefit calculations in this paradox, our results show that the psychological and relational benefits of self-disclosure, such as enhanced trust and reduced loneliness, may serve as important motivations that override privacy concerns. This insight contributes to a more balanced understanding of why users continue to engage in self-disclosure online.

Beyond theoretical insights, this study opens possibilities for future interdisciplinary exploration. For example, further research can investigate how platform design elements (e.g., visibility controls, content types) influence users’ willingness to self-disclose and their social capital accumulation. Moreover, future studies might consider cross-cultural comparisons or longitudinal designs to better understand the long-term impacts of online self-disclosure. These directions could further support the development of healthy and trustful online environments in both academic and applied contexts.

## Figures and Tables

**Table 1:** Descriptive statistics and correlations.

	M	SD	1	2	3	4	5	6	7	8
1. Gender	-	-								
2. Age	21.63	1.09	0.26 **							
3. Average daily online time	6.81	2.82	0.02	−0.02						
4. Social media self-disclosure	3.22	1.25	0.19 ***	0.10 ***	0.01					
5. Online bridging social capital	3.43	0.65	−0.01	−0.01	0.07 **	0.40 ***				
6. Online bonding social capital	2.58	0.67	0.17 ***	0.0 **	−0.01	0.41 ***	0.36 ***			
7. Loneliness	2.12	0.46	0.15 ***	0.00	0.064	−0.04	−0.20 ***	0.02		
8. Online interpersonal trust	2.52	0.52	0.21 ***	0.11 ***	−0.03	0.43 ***	0.21 ***	0.43 ***	0.04	
9. Agreeableness	3.22	0.38	0.03	0.10 ***	0.04	0.13 ***	0.17 ***	0.00	−0.18 ***	0.09 ***

Note: *N* = 1519. ** *p* < 0.01. *** *p* < 0.001.

**Table 2:** Testing the mediation effect of bridging online social capital on loneliness.

Predictors	Model 1 (Online Bridging Social Capital)			Model 2 (Loneliness)		
	*β*	*SE*	*t*	*β*	*SE*	*t*
Gender	−0.12	0.04	−2.83 *	0.18	0.03	5.82 ***
Age	−0.01	0.02	−0.81	−0.02	0.01	−1.46
Average daily online time	0.02	0.01	2.96	0.01	0.00	1.89
Social media self-disclosure	0.21	0.01	16.32 ***	0.00	0.02	0.14
Online bridging social capital				−0.15	0.02	−7.44 ***
Constant	2.92	0.33	8.95 ***	2.89	0.25	11.46 ***
R^2^	0.16			0.07		
F	68.88 ***			20.49 ***		

Note: *N* = 1519. * *p* < 0.05. *** *p* < 0.001.

**Table 3:** Testing the mediation effect of bonding online social capital on online interpersonal trust.

Predictors	Model 3 (Online Bonding Social Capital)			Model 4 (Online Interpersonal Trust)		
	*β*	*SE*	*t*	*β*	*SE*	*t*
Gender	0.16	0.04	3.79 ***	0.12	0.03	3.85 ***
Age	0.01	0.02	0.81	0.01	0.01	1.17
Average daily online time	−0.00	0.01	−0.76	−0.01	0.00	−1.30
Social media self-disclosure	0.21	0.01	16.10 ***	0.12	0.01	11.06 ***
Online bonding social capital				0.23	0.02	11.92 ***
Constant	1.62	0.33	4.88 ***	1.29	0.24	5.34 ***
R^2^	0.18			0.27		
F	79.14 ***			105.75 ***		

Note: *N* = 1519. *** *p* < 0.001.

**Table 4:** Testing the moderation effect of agreeableness.

	Model 5 (Online Bridging Social Capital)			Model 6 (Loneliness)			Model 7 (Online Bonding Social Capital)			Model 8 (Online Interpersonal Trust)		
	*β*	*SE*	*t*	*β*	*SE*	*t*	*β*	*SE*	*t*	*β*	*SE*	*t*
Gender	−0.12	0.04	−2.85 **	0.19	0.03	5.92 ***	0.16	0.04	3.79 ***	0.12	0.03	3.84 ***
Age	−0.02	0.01	−1.34	−0.02	0.01	−1.48	0.01	0.02	0.91	0.01	0.01	1.21
Average daily online time	0.02	0.01	3.07 **	0.01	0.00	2.06 *	−0.00	0.01	−0.70	−0.01	0.00	−1.27
Social media self-disclosure	0.10	0.10	0.99	−0.00	0.01	−0.10	−0.07	0.11	−0.63	0.11	0.01	10.88 ***
Agreeableness	0.10	0.12	0.80				−0.41	0.13	−3.79 ***			
Social media self-disclosure × Agreeableness	0.03	0.03	1.05				0.09	0.03	2.70 **			
Online bridging social capital				−0.14	0.02	−7.12 ***						
Online bonding social capital										0.23	0.02	11.77 ***
Constant	2.79	0.50	5.58 ***	2.88	0.25	11.36 ***	2.91	0.51	5.66 ***	1.29	0.24	5.30 ***
R^2^	0.19			0.07			0.19			0.27		
F	52.30 ***			20.07 ***			53.83 ***			102.31 ***		

Note: *N* = 1519. * *p* < 0.05. ** *p* < 0.01. *** *p* < 0.001.

## Data Availability

The original contributions presented in this study are included in the article. Further inquiries can be directed to the corresponding author.

## Appendix A. The Survey

### Appendix A.1. Demographic Information

- What is your gender?

1. Male 2. Female

- What is your year of birth?

(Please enter a 4-digit number, e.g., 1998): _______

- On average, how many hours do you spend online per day?

(Please enter a whole number): _______ hours

### Appendix A.2. Loneliness

- I feel in tune with the people around me.
- I lack companionship.
- There is no one I can turn to.
- I do not feel alone.
- I feel part of a group of friends.
- I have a lot in common with the people around me.
- I am no longer close to anyone.
- My interests and ideas are not shared by those around me.
- I am an outgoing person.
- There are people I feel close to.
- I feel left out.
- My social relationships are superficial.
- No one really knows me well.
- I feel isolated from others.
- I can find companionship when I want it.
- There are people who really understand me.
- I am unhappy being so withdrawn.
- People are around me but not with me.
- There are people I can talk to.
- There are people I can turn to.

### Appendix A.3. Online Interpersonal Trust

- I believe most users provide truthful information online.
- After interacting with someone online for a while, I tend to feel that they are trustworthy.
- The personal information I provide online is truthful.
- Most people online are generally honest and reliable.
- If you’re not cautious online, others may take advantage of you.
- In online interpersonal interactions, people are generally trustworthy.
- I can confide my secrets to online friends, and I know they are willing to listen.
- Online, my friends’ behavior is unpredictable and hard to grasp; sometimes I can’t be sure how they will act.

### Appendix A.4. Online Social Capital

- There are several people on social media I trust to help solve my problems.
- There is someone on social media I can turn to for advice about making very important decisions.
- There is no one on social media that I feel comfortable talking to about intimate personal problems.
- When I feel lonely, there are several people on social media I can talk to.
- If I needed an emergency loan of $500, I know someone on social media I can turn to.
- The people I interact with on social media would put their reputation on the line for me.
- The people I interact with on social media would be good job references for me.
- The people I interact with on social media would share their last dollar with me.
- I do not know people on social media well enough to get them to do anything important.
- The people I interact with on social media would help me fight an injustice.
- Interacting with people on social media makes me interested in things that happen outside of my town.
- Interacting with people on social media makes me want to try new things.
- Interacting with people on social media makes me interested in what people unlike me are thinking.
- Talking with people on social media makes me curious about other places in the world.
- Interacting with people on social media makes me feel like part of a larger community.
- Interacting with people on social media makes me feel connected to the bigger picture.
- Interacting with people on social media reminds me that everyone in the world is connected.
- I am willing to spend time to support general social media community activities.
- Interacting with people on social media gives me new people to talk to.
- Social media, I come in contact with new people all the time.

### Appendix A.5. Social Media Self-Disclosure

- When I have something to say, I enjoy sharing it on social media.
- I am willing to let my friends know what’s going on in my life through social media.
- I provide detailed and comprehensive personal information on my social media profile.
- I keep my personal profile up to date on social media.
- I disclose personal matters publicly on social media.
- I express my feelings, emotions, and experiences on social media in an honest and in-depth way.

### Appendix A.6. Big Five Personality Scale

1. Bashful	1 2 3 4 5	2. Moody	1 2 3 4 5
3. Bold	1 2 3 4 5	4. Organized	1 2 3 4 5
5. Careless	1 2 3 4 5	6. Philosophical	1 2 3 4 5
7. Cooperative	1 2 3 4 5	8. Quiet	1 2 3 4 5
9. Creative	1 2 3 4 5	10. Relaxed	1 2 3 4 5
11. Cold	1 2 3 4 5	12. Rude	1 2 3 4 5
13. Complex	1 2 3 4 5	14. Shy	1 2 3 4 5
15. Deep	1 2 3 4 5	16. Sloppy	1 2 3 4 5
17. Disorganized	1 2 3 4 5	18. Sympathetic	1 2 3 4 5
19. Efficient	1 2 3 4 5	20. Practical	1 2 3 4 5
21. Energetic	1 2 3 4 5	22. Systematic	1 2 3 4 5
23. Envious	1 2 3 4 5	24. Talkative	1 2 3 4 5
25. Extraverted	1 2 3 4 5	26. Temperamental	1 2 3 4 5
27. Fretful	1 2 3 4 5	28. Touchy	1 2 3 4 5
29. Harsh	1 2 3 4 5	30. Uncreative	1 2 3 4 5
31. Imaginative	1 2 3 4 5	32. Unenvious	1 2 3 4 5
33. Inefficient	1 2 3 4 5	34. Unintellectual	1 2 3 4 5
35. Intellectual	1 2 3 4 5	36. Unsympathetic	1 2 3 4 5
37. Jealous	1 2 3 4 5	38. Warm	1 2 3 4 5
39. Kind	1 2 3 4 5	40. Withdrawn	1 2 3 4 5
